# Preparation and Properties of Self-Healing and Self-Lubricating Epoxy Coatings with Polyurethane Microcapsules Containing Bifunctional Linseed Oil

**DOI:** 10.3390/polym11101578

**Published:** 2019-09-27

**Authors:** Haijuan Yang, Qiufeng Mo, Weizhou Li, Fengmei Gu

**Affiliations:** 1 School of Resources, Environment and Materials, Guangxi University, Nanning 530004, China; yanghaijuan2019@163.com (H.Y.); 18078131362@163.com (Q.M.); gufengmei32@163.com (F.G.); 2 Guangxi Key Laboratory of Processing for Non-ferrous Metals and Featured Materials, Guangxi University, Nanning 530004, China

**Keywords:** coating, microcapsules, linseed oil, self-healing, self-lubricating

## Abstract

An organic coating is commonly used to protect metal from corrosion, but it is prone to failure due to microcracks generated by internal stress and external mechanical action. The self-healing and self-lubricating achieved in the coating is novel, which allows an extension of life by providing resistance to damage and repair after damage. In this study, a new approach to microencapsulating bifunctional linseed oil with polyurethane shell by interfacial polymerization. Moreover, the self-healing and self-lubricating coatings with different concentrations of microcapsules were developed. The well-dispersed microcapsules showed a regular spherical morphology with an average diameter of ~64.9 μm and a core content of 74.0 wt.%. The results of the salt spray test demonstrated that coatings containing microcapsules still possess anticorrosion, which is improved with the increase of microcapsules content, after being scratched. The results of electrochemical impedance spectroscopy showed a |Z|_f=0.01Hz_ value of 10^4^ Ω·cm^2^ for pure epoxy coating after being immersed for 3 days, whereas the coating with 20 wt.% microcapsules was the highest, 10^10^ Ω·cm^2^. The results of friction wear showed that the tribological performance of the coating was enhanced greatly as microcapsule concentration reached 10 wt.% or more, which showed a 86.8% or more reduction in the friction coefficient compared to the pure epoxy coating. These results indicated that the coatings containing microcapsules exhibited excellent self-healing and self-lubricating properties, which are positively correlated with microcapsules content.

## 1. Introduction

Corrosion causes huge economic losses every year in various fields, such as marine, construction, and aerospace [1,2]. Organic coating can block the migration passage of water and oxygen to the metal surfaces, and it is commonly used to protect metal from corrosion [3,4]. Nonetheless, during its preparation and use, the coating is prone to microcracks inside the structure due to solvent evaporation, mechanical action, temperature change, etc. [1,5,6]. As the crack expands, the metal is contacted with the corrosive medium and the coating loses its protective ability. Furthermore, the initial microcracks are very difficult to detect and repair. So, the development of alternative protections is extremely urgent.

Self-healing coatings have been considered as a promising method to address the above challenges, due to the automatic damages healing without external intervention [7,8]. White et al. [9] first encased dicyclopentadiene (DCPD) inside urea–formaldehyde (UF) shells via in situ polymerization to form microcapsules, and found that the epoxy matrix containing microcapsules and Grubbs catalyst showed self-healing after damage. After that, loading microcapsules with healing agents to develop self-healing coating has attracted great interest [10,11]. In addition to DCPD, various healing agents have been microencapsulated for self-healing systems, including epoxy [12,13,14], polydimethylsiloxane [15,16,17], dicyclohexylmethane diisocyanate (HMDI) [18], hexamethylene diisocyanate (HDI) [19], isophorone diisocyanate (IPDI) [20,21,22,23], Polyaryl polymethylene isocyanates (PAPI) [24], tung oil (TO) [25], linseed oil (LO) [6], etc. Among them, LO is a good candidate for healing agents because it is cheap, environmentally friendly, and air-drying, which can be polymerized with oxygen into a flexible and water-repellent coating [26,27,28]. Moreover, the lubricity of LO has been reported. Kozdrach et al. [29] have shown the impact of polyvinylpyrrolidone on friction and wear performances of LO-based grease. LO has a similar viscosity and thermal stability to lubricate oil, which make it a prominent lubricant. This means tLO can act as a bifunctional agent for self-healing and self-lubricating. Poly(urea–formaldehyde) microcapsules containing TO were studied to heal and resist wear [30]. HMDI-filled microcapsules has been reported as the healing and lubricating agent [18]. The self-healing and self-lubricating achieved in the coating is novel, which allows an extension of life by providing resistance to damage and repair after damage. So far, most studies have concentrated on single-function coatings, whereas dual-function coatings with self-healing and self-lubricating have rarely been reported [18,30,31]. The multifunctional characteristics achieved in the coating matrix are the focus of smart coatings in the future. Developing smart coatings on self-healing and self-lubricating properties is extremely challenging [18]. Therefore, LO was selected as the core material for this study.

Suryanarayana et al. [6] encased LO in urea formaldehyde (UF) shells through in situ polymerization, and found that LO could heal the coating to prevent metal corrosion. Szabó et al. [32] loaded LO, co-octoate, and octadecylamine (ODA) within UF microcapsules to recover damage and obtain a better anticorrosion ability. Hasanzadeh et al. [5] applied UF to encase LO and CeO_2_ nanoparticles, investigating the self-healing ability of coatings with these microcapsules. UF is commonly used to encapsulate LO via in situ polymerization in most studies. UF resin is generally formed by reacting urea with formaldehyde in a stoichiometric ratio. Moreover, UF resin may be degraded to release formaldehyde if it is affected by temperature, humidity, pH and chemicals during use [33]. It is accompanied by safety hazards during use. It is urgent to develop ecofriendly shell materials to load LO microcapsules. Therefore, polyurethane was used as the shell material of the microcapsules in this study.

In this study, we encapsulated LO into polyurethane shells instead of unfriendly UF shells. The polyurethane shell was formed by interfacial polymerization using TMP and polyisocyanate. The prepared LO-loaded microcapsules were embedded into epoxy coating for investigation of their self-healing and self-lubricating properties. Scanning electron microscopy (SEM), Fourier transform infrared spectroscopy (FTIR), and thermogravimetric analysis (TGA) were used to characterize the properties of the microcapsules. Salt spray tests, electrochemical impedance spectroscopy (EIS), and friction wear test were carried to evaluate the coating on the self-healing and self-lubricating properties.

## 2. Materials and Methods 

### 2.1. Materials

Epoxy resin Epikoe 862, thinner Heloxy 8, and related hardener Epikure 205 were obtained from Hexion Specialty Chemicals. (Columbus OH, America). LO serves as the core material and was procured from Damao Chemical Reagent Factory (Tianjin, China). Desmodur L-75 with an isocyanate group (NCO) content of 13.3 ± 0.4 wt.%, used as a material for preparing the shell, was purchased from Bayer MaterialScience (Leverkusen, Germany). 1,1,1-trimethylolpropane (TMP) and gum arabic (GA) were purchased from Aladdin (Shanghai, China). Ethyl acetate (EtOAc) was used as solvent and obtained from Guangdong Guanghua Sci-Tech Co.,Ltd. (Guangdong, China) The above chemicals were utilized without further purification.

### 2.2. Synthesis of PU-Encapsulated LO Microcapsules

Polyurethane (PU) microcapsules containing LO were prepared in two steps by interfacial polymerization. In the first step, an organic phase was carried out by dissolving LO (24.0 g) and L-75 (8.0 g) into EtOAc (20.0 g). A mixture was prepared by mixing the organic phase and aqueous emulsifier solution, which was provided by dissolving GA (12.0 g) into 120.0 g of deionized water. The mixture was emulsified at 1200 rpm for 5 min to acquire stable emulsion system. In the second step, polymerization reaction was started by adding a solution containing 4.0 g of TMP and 20.0 g deionized water to the emulsion system dropwise. The reaction system was heated to 50 °C for 1.5 h with continuously stirring at 400 rpm to complete the formation of the microcapsules. The suspended microcapsules were separated after placing the reacted solution for 2 h. The microcapsules were filtered under negative pressure with deionized water for several times and then dried in the environment for 24 h.

### 2.3. Preparation of Epoxy Composites 

Epoxy resin was obtained by formulating Epikoe 862, Epikure F 205, and Heloxy 8 at mass ratio of 1:0.58:0.1. Microcapsules were added in the epoxy resin at different concentrations is 5, 10, 15, and 20 wt.%. The pure epoxy sample without microcapsules was prepared as a control sample.

**Figure 1:**
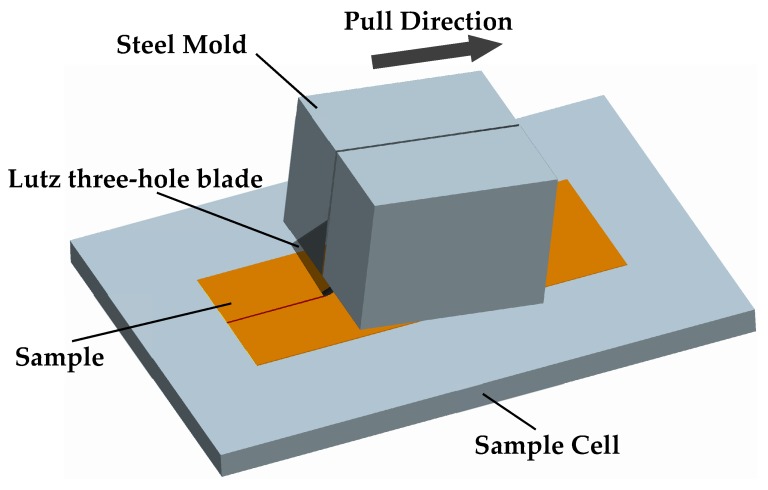
Schematic diagram of the homemade scratch apparatus.

The samples for salt spray test and EIS measurements were prepared via applying a coating having a dry thickness of 250 ± 20 μm on steel plate (120 × 50 × 1 mm^3^) with a drawdown bar. After all samples were cured at room temperature for 24 h, cross scratches deep into the substrate were created on samples using a homemade scratch apparatus (Figure 1). Scratched samples were placed at room temperature for 5 days and then subjected to thermal curing at 80 °C for 4h to ensure the completion of self-healing [34], followed by salt spray and EIS tests.

The samples for friction and wear tests were fabricated by placing epoxy composites to a cylindrical silicone mold with a diameter of 30 mm. After 24 h for curing, the samples were removed from the mold. The test surfaces of all samples were treated with 2000 mesh sandpaper then cleaned with ethanol prior to the friction test.

### 2.4. The Characterization of Microcapsules

The FTIR spectra of the microcapsules, LO, polyurethane resin, and the extracted core were recorded by using FTIR spectrometer (Frontier, Perkin Elmer, Shanghai, China) in attenuated total reflectance (ATR) mode with the range of 4000 to 650 cm^−1^. The core material was separated from broken microcapsules by centrifugation with EtOAc. 

The TG curves of microcapsules, LO and polyurethane resin were recorded by using thermogravimetric analyzer (DTG-60(H), SHIMADZU, Kyoto, Japan) in N_2_ environment. The sample mass was approximately 5 mg and the test temperature range was 40 to 600 °C with a heating rate of 10 °C/min.

The morphology of microcapsules was observed by a scanning electron microscope (SEM, SU8020, Hitachi). Microcapsules were spread on conductive tape and sputtered with gold. To observe the internal structure, some of the microcapsules were intentionally broken. 

The size distribution of microcapsules was measured by a Mastersizer particle size analyzer (Mastersizer 2000, Malvern). 

### 2.5. The Characterization of Coatings with Microcapsules

The corrosive protection of the coating to metal substrates after being scratched was evaluated by neutral salt spray test. Prior to the test, the fringe and back of all samples were covered with paraffin wax. The samples were exposed to 5 wt.% of NaCl fog at 35 ± 1 °C for 1, 2, 3, and 4 weeks according to ISO 9227-2017. 

The corrosion resistance behavior of the scratched coating in 3.5 wt.% NaCl solution was evaluated by EIS. EIS measurement was performed on the electrochemistry workstation (CHI750E, Shanghai, China) via a three-electrode system. The reference electrode, counter electrode, and working electrode were assumed by saturated calomel electrode (Hg/Hg_2_Cl_2_/KCl), platinum foil, and the sample with an exposed district of 9.07 cm^2^, respectively. A frequency range of 10^-2^ to 10^5^ Hz and an AC amplitude of 10 mV were selected. The software Zview was carried to fit the EIS results. 

The self-lubricating ability of the coating was evaluated by a linear reciprocating ball-on-disk friction wear tests performed on a tribometer (UMT-TriboLab, BRUKER, Milan, Italy) at ambient temperature. The samples were used to test disks and the GCr15 steel ball (Φ4 mm) was taken as the counterpart. The test parameters are shown below: Applied load: 3 N; stroke: 5mm; sliding frequency: 5 Hz; velocity: 5 cm/s; test time: 30 min. The friction coefficient of all samples was measured for analysis. The wear tracks were obtained through Nexview optical profilometer (ZYGO). The morphology of wear tracks was imaged through SEM.

## 3. Results and Discussion

### 3.1. Synthesis and Morphology of Microcapsules

**Scheme 1:**
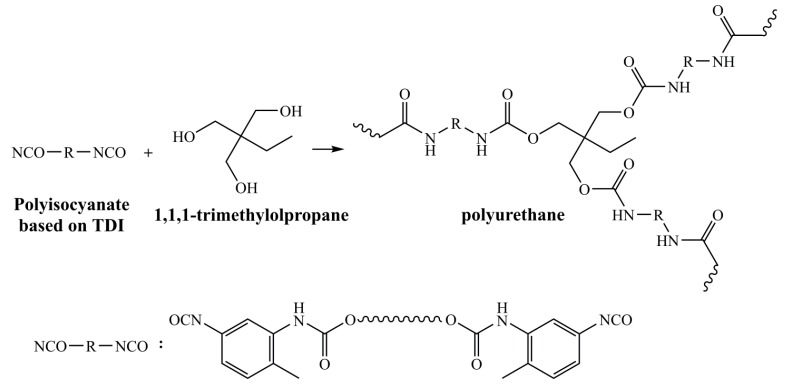
Reaction scheme for shell formation of polyurethane microcapsules.

**Figure 2:**
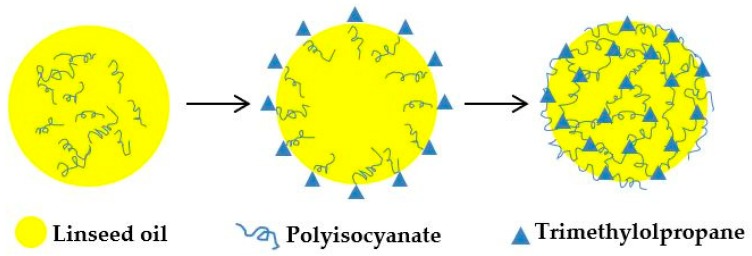
Schematic illustration of the microcapsules formation.

Polyurethane microcapsules containing LO were synthesized via interfacial polymerization reaction occurred in an oil in water emulsion. L-75 is a TDI-based polyisocyanate that has previously been reported to encapsulate IPDI [35]. Trifunctional TMP dissolved in the aqueous phase as a cross-linking agent was favorable for higher cross-link density in the polymer structure. The OH of TMP and the NCO of L75 polymerize at the surfaces of microdroplets to form polyurethane, thereby encapsulating LO. GA acts as an emulsifier to allow the PU to deposit slowly on the surface of the microdroplets [35,36]. Feasible reaction mechanism for shell formation of polyurethane microcapsules is shown in Scheme 1. Figure 2 illustrates a schematic for the fabrication of polyurethane microcapsules containing LO via interfacial polymerization.

**Figure 3:**
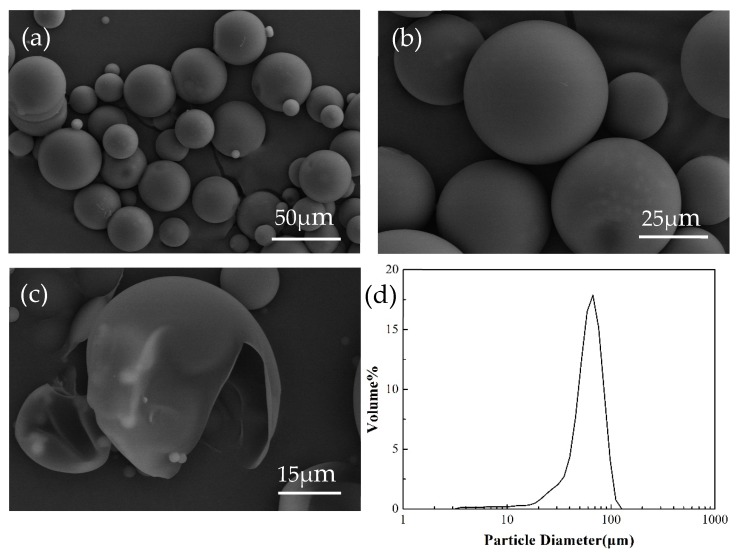
The morphology of linseed oil (LO) microcapsules: (**a**) overview of microcapsules with distributed diameters, (**b**) microcapsules magnification, (**c**) hollow structure of microcapsules, and (**d**) distribution of diameter of microcapsules.

Microcapsules morphologies and particle size distribution were presented in Figure 3. It observes that the microcapsules present regular spherical shapes. Moreover, there is no adhesion between microcapsules, which is advantageous for their dispersion in the resin. Microcapsules show a smooth and compact surface, without holes and pits, indicating that the strength of microcapsules is sufficient to withstand the inevitable mechanical collision in preparation and use. It also provides a barrier to prevent LO from reacting with oxygen. A distinct hollow internal structure can be observed after microcapsules were crushed. The average diameter of microcapsules is 64.9 μm (Figure 3d). 

### 3.2. FTIR Spectroscopy of Microcapsules

**Figure 4:**
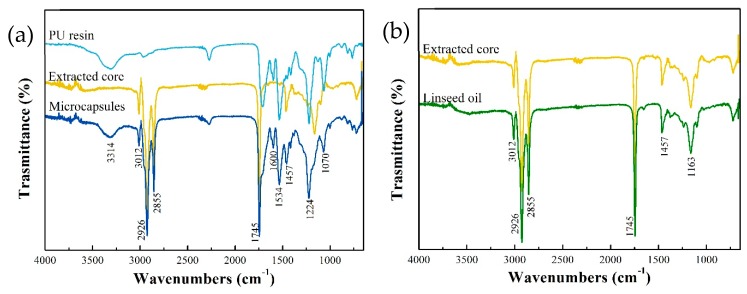
(**a**) FTIR spectra of Polyurethane (PU) resin, LO, and microcapsules containing core material. (**b**) FTIR spectra of extracted core and LO.

The composition of the prepared microcapsules was analyzed by FTIR analysis. Figure 4 shows the FTIR spectrums of PU resin, LO, microcapsules, and the extracted core, respectively. The absorption bands of urethane groups (NHCOO) are exhibited in the spectrum: N–H stretching appears at 3314 cm^−1^, C=O stretching at 1715 cm^−1^, N–H bending at 1534 cm^−1^, C–N stretching at 1224 cm^−1^, and C–O stretching at 1070 cm^−1^ [35]. The stretching vibration of the benzene ring is clearly observed at 1600 cm^−1^, which confirmed the synthesis of PU resin. The spectrum of LO displayed the absorption bands of unsaturated C–H stretching at 3012 cm^−1^, saturated C–H stretching at 2926 cm^−1^, saturated C–H stretching at 2855 cm^−1^, C=O stretching at 1745 cm^−1^, C–H bending at 1457 cm^−1^, and C–COO stretching at 1162 cm^−1^ [27]. The absorption bands characteristics of PU resin and LO are conjointly presented in the spectrum of microcapsules. In addition, the core material was separated from broken microcapsules to further confirm the encapsulation of LO, and its FTIR spectrums as showed in Figure 4b. The spectrum of the LO matched the extracted core at the characteristic peak, confirming that LO was successfully encapsulated by PU shell.

### 3.3. Thermal Stability of Microcapsules

**Figure 5:**
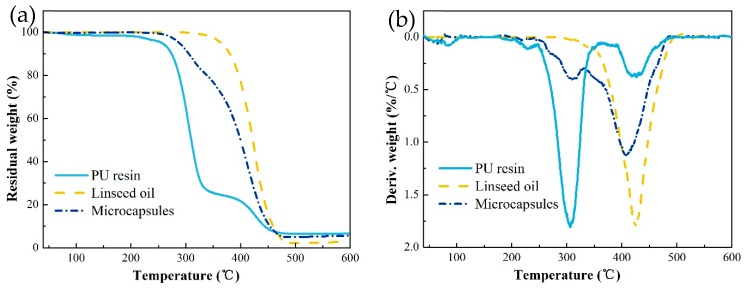
Thermal gravimetric (TG) (**a**) and differential thermogravimetric (DTG) (**b**) of LO, PU resin, and microcapsules containing core material.

To determine the service temperature range and core content of microcapsules, its thermal properties were investigated by TGA. The TG curves of PU resin, LO, and microcapsules are exhibited in Figure 5a. The first derivative of TG curves to temperature is showed in Figure 5b. LO underwent decomposition in the range of 300 to 480 °C, and its maximum decomposition rate appears at 426 °C, indicating better thermal stability compare to frequently used core materials such as epoxy [14], HMDI [18], and IPDI [22]. The PU shell is initially degraded at 250 °C and ended at 470 °C, achieving a maximum decomposition rate at 306 °C. It can be concluded that the weight loss trend of the microcapsules is consistent with that of PU below 300 °C. With the temperature rising to 300 °C, a significant decrease in the mass of microcapsules was observed for the joint decomposition of the PU shell and the LO. Thermal stability of microcapsules is between the PU shell and LO. Accordingly, we can conclude that the microcapsules encapsulating LO was successfully fabricated. And PU microcapsules are more thermally stable than UF microcapsules, which experience a weight loss at ~100–200 °C due to the release of free formaldehyde and formaldehyde split from ether groups [37,38,39,40].

The core content of microcapsules is calculated by the TG curves. There is nearly no mass loss of LO at 300 °C, and the mass loss of microcapsules at the corresponding temperature can be attributed entirely to the decomposition of PU shell. Therefore, the ratio of the mass loss of microcapsules to PU shell equals to the shell content, and the core content is correspondingly obtained. The core content of microcapsules is calculated as high as 74 wt.%, indicating that the encapsulation is effective.

### 3.4. Salt Spray Test

**Figure 6:**
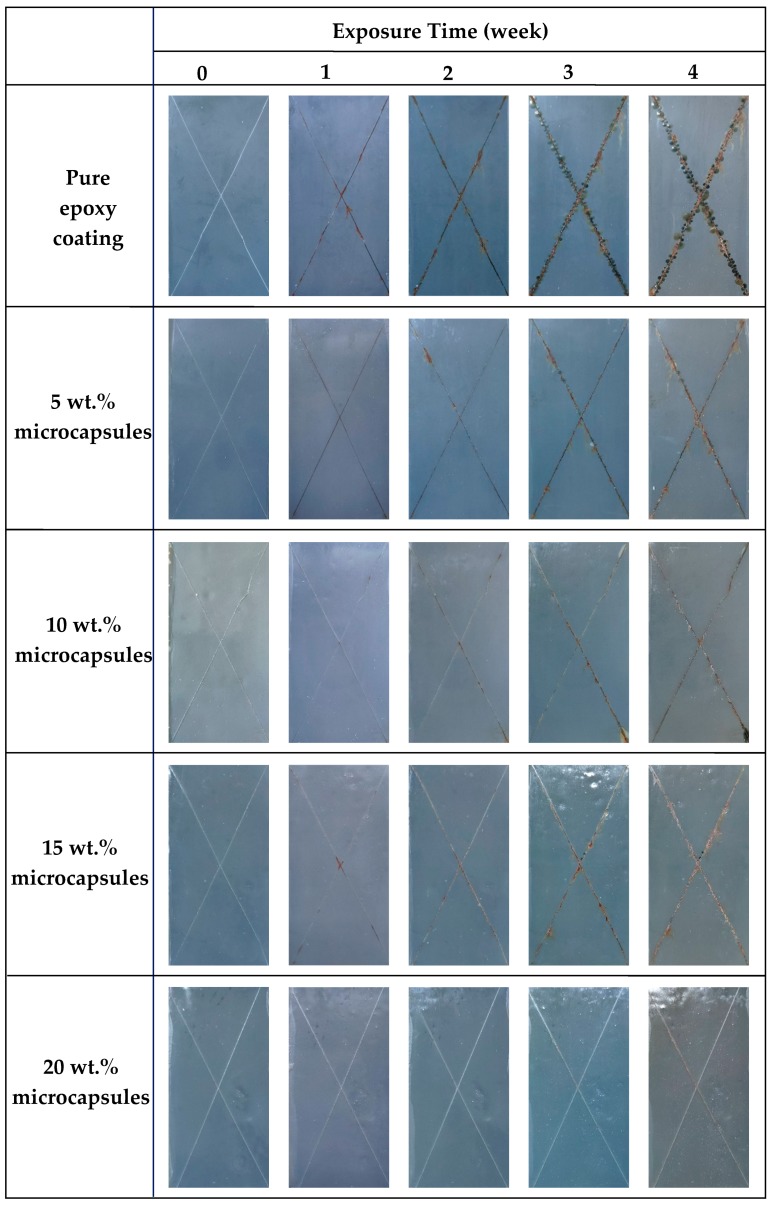
Salt spray test results for pure epoxy coatings and self-healing coatings containing 5, 10, 15, and 20 wt.% microcapsules after 0, 1, 2, 3, and 4 weeks exposure.

The systematic neutral salt spray tests were conducted to evaluate the self-healing property of the coating containing LO-loaded microcapsules. The pure epoxy coating and the coatings with microcapsules were exposed to salt spray for 0, 1, 2, 3, and 4 weeks, and the results are shown in Figure 6. The rust phenomenon became more serious as the exposure time increases, and the corrosion resistance of coatings improved as the increase of microcapsules content. After 1 week exposure, obvious rust marks appeared in the scratched areas of the pure epoxy sample. In comparison, coatings with microcapsules showed slight rust. After 4 weeks, the scratched areas of the pure epoxy sample have been completely covered by corrosion products and obvious rust blisters showed around it. The coatings with microcapsules exhibit varying degrees of corrosion that is far less visible than the pure epoxy. Among them, the coating containing 20 wt.% microcapsules displayed the best corrosion protection to steel substrate, which showed no evident rust 4 weeks after explosion. Therefore, the coating containing microcapsules have self-healing performance and it is positively correlated with microcapsule content. This may be due to the higher the content of microcapsules, the more LO is used to fill the scratched areas and provide a denser barrier.

**Figure 7:**
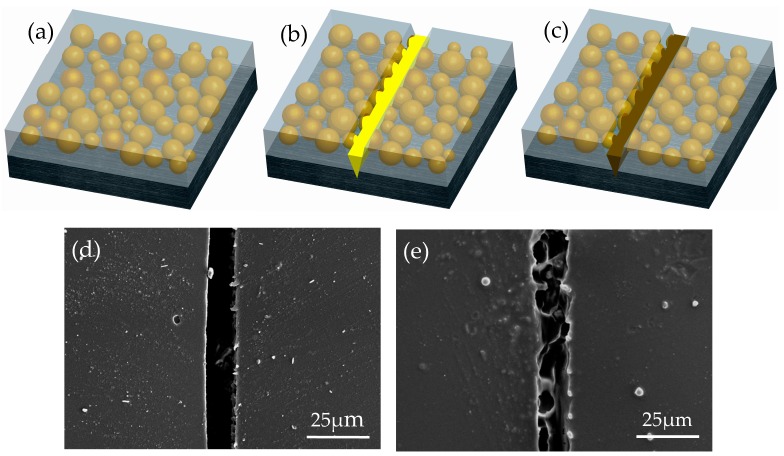
Scheme of the self-healing process. (**a**) Self-healing coating containing microcapsules on a metallic substrate. (**b**) Damage of the coating; release of the encapsulated LO on the metal surface. (**c**) A crack healed by the polymerization of LO with oxygen. SEM micrographs of the scratched area of the (**d**) pure epoxy coating and (**e**) self-healing coating with 10 wt.% microcapsules.

To further confirm the self-healing of coating containing microcapsules, the scratched area in pure epoxy coating and the coating with 10 wt.% microcapsules were observed by SEM. Artificial scratch of the coating with 10 wt.% microcapsules is covered by a new film, whereas that of the pure epoxy coating is not observed, as shown in Figure 7. After the coating containing microcapsules is damaged, the encapsulated LO is released to the microcracks by capillary action and polymerized with oxygen to heal the crack. The scheme of the self-healing procedure is indicated in Figure 7a–c. It provides a new barrier, while the pure epoxy keeps the scratched portion bare. Obviously, the leakproofness of this new barrier is greatly affected by the microcapsules content.

### 3.5. EIS Measurement

**Figure 8:**
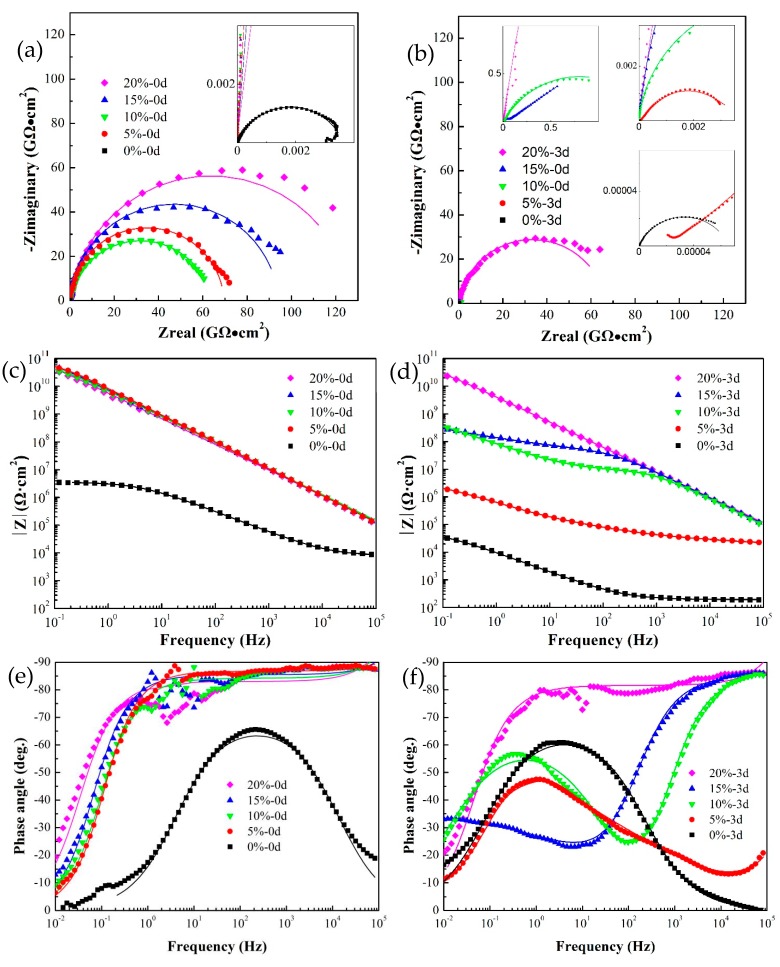
Nyquist plots (**a,b**), Bode impedance (**c,d**), and Bode phase (**e,f**) of pure epoxy coatings and self-healing coatings containing 5, 10, 15, and 20 wt.% microcapsules after scratching and immersing in 3.5% NaCl electrolyte for 0 (a,c,e) and 3 (b,d,f) days. The points represent experimental data and the continuous lines with the same color represent the fits.

The self-healing of coatings with 5, 10, 15, and 20 wt.% microcapsules was further evaluated with EIS technique. The Nyquist and Bode diagrams are displayed in Figure 8. The equivalent circuit is equipped with the constant phase element *Q* (CPE), which is calculated by the following formula [41],

*Z_Q_ = T^*−1*^(jω)^−P^*, (1)

where *T* is a constant with dimension F·cm^−2^s^P−1^, *j = *(−1)*^*1/2*^*, *ω* is the angular frequency, and *P* is the exponent related to the degree of frequency dispersion.

**Figure 9:**
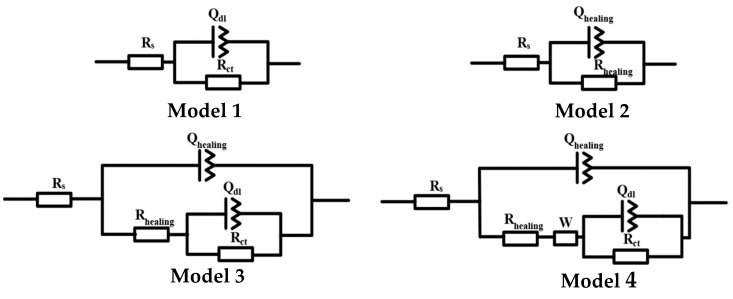
Four models of equivalent circuits.

The equivalent circuit models were shown in Figure 9. The circuit model parameters were presented in Table 1. *R_s_* is the solution resistance. *Q_dl_* and *R_ct_* are the electric double-layer capacitor and charge-transfer resistance. *Q_healing_* and *R_healing_* represent the healing resistance and healing capacitor. *W* is the Warburg impedance.

After immersion for 0 days, a single capacitive loop of pure epoxy was observed in the corresponding Nyquist plot. The electrochemical response spectra of pure epoxy sample shows the one time constant, and it was fitted with model 1. In comparison, the EIS results of coatings with 5, 10, 15, and 20 wt.% microcapsules were fitted with model 2, taking into account the healing capacitance and healing resistance. Their radius of single capacitive loop (*R_healing_*) were above 10^10^ Ω·cm^2^, much higher than the pure epoxy (*R_ct_* = 3.62 × 10^6^ Ω·cm^2^). Correspondingly, the Bode plots show higher impedance modulus (*|Z|*) in the 10^−2^ to 10^5^ Hz range for the coatings with microcapsules than the pure epoxy coating. Moreover, the phase angle of the coatings with microcapsules is close to −90° over a wide range, which means that the coatings with microcapsules have good protection performance after being scratched.

After immersion for 3 days, the pure epoxy sample shows a decrease in the value of *|Z|_f = 0.01Hz_* to 10^4^ Ω·cm^2^. Besides, the Bode phase plots show the single peak moving to a lower frequency. It indicated that the pure epoxy sample takes a further reduction in corrosion resistance. The *|Z|_f = 0.01Hz_* of the coating with 5 wt.% microcapsules dropped sharply to 10^6^ Ω·cm^2^. Furthermore, the Warburg impedance characteristics are presented in the intermediate frequency, and the EIS result was fitted with model 4. This indicated that the coating has continuous diffusion channels for corrosive medium, and its corrosion protection performance is basically ineffective but still better than the pure epoxy sample. The electrochemical response of the coating with 10 and 15 wt.% microcapsules transitioned to double capacitive loop, illustrating that the electrolyte solution has penetrated into the metal interface through the weak point of the healing layer and the corrosion reaction has begun. The EIS results of them were fitted by Model 3. However, the coating with 15wt.% microcapsules has better corrosion resistance, since the capacitive loop at high frequency shows a larger diameter and the capacitive loop in low frequency is not fully formed. Model 2 is still suitable for the EIS results of the coating with 20 wt.% microcapsules, *R_healing_* only dropped from 10^11^ to 10^10^ Ω·cm^2^, maintaining good corrosion resistance. In conclusion, he coating with microcapsules exhibited self-healing ability, which was positively correlated with the microcapsules content. This is in agreement with the salt spray test results.

### 3.6. Self-Lubricating Mechanism

**Figure 10:**
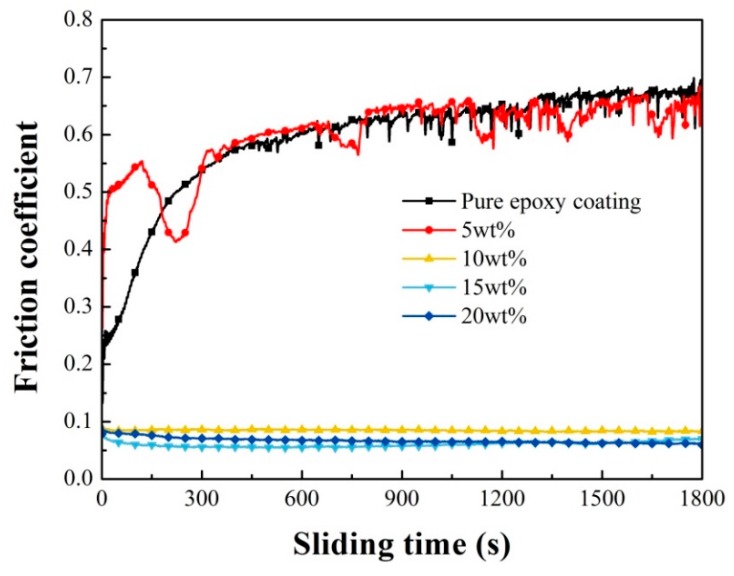
Friction coefficients of pure epoxy coating and the coatings with 5, 10, 15, and 20 wt.% microcapsules as a function of sliding time.

**Figure 11:**
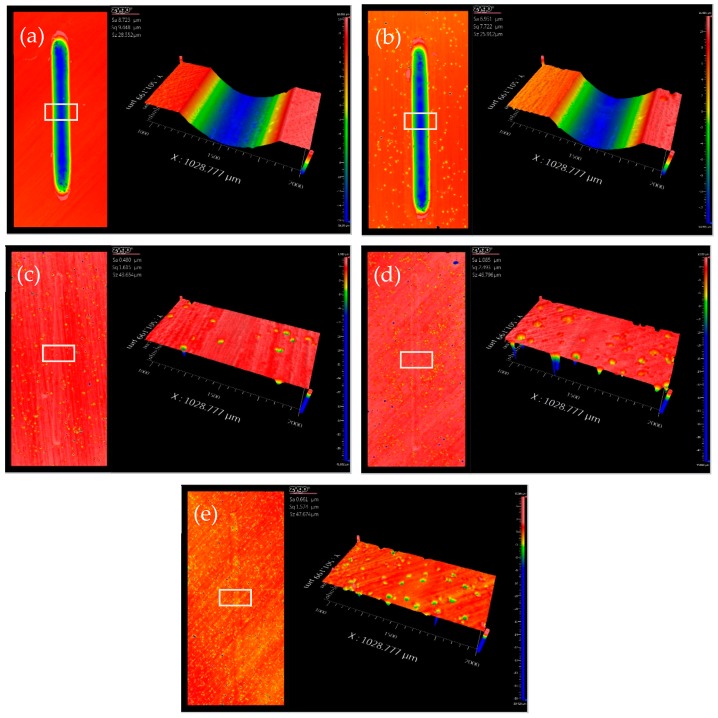
The topographies of samples after tribological tests containing (**a**) a pure epoxy coating and (**b**–**e**) a coating with 5, 10, 15, and 20 wt.% microcapsules and the respective partial enlargement.

**Figure 12:**
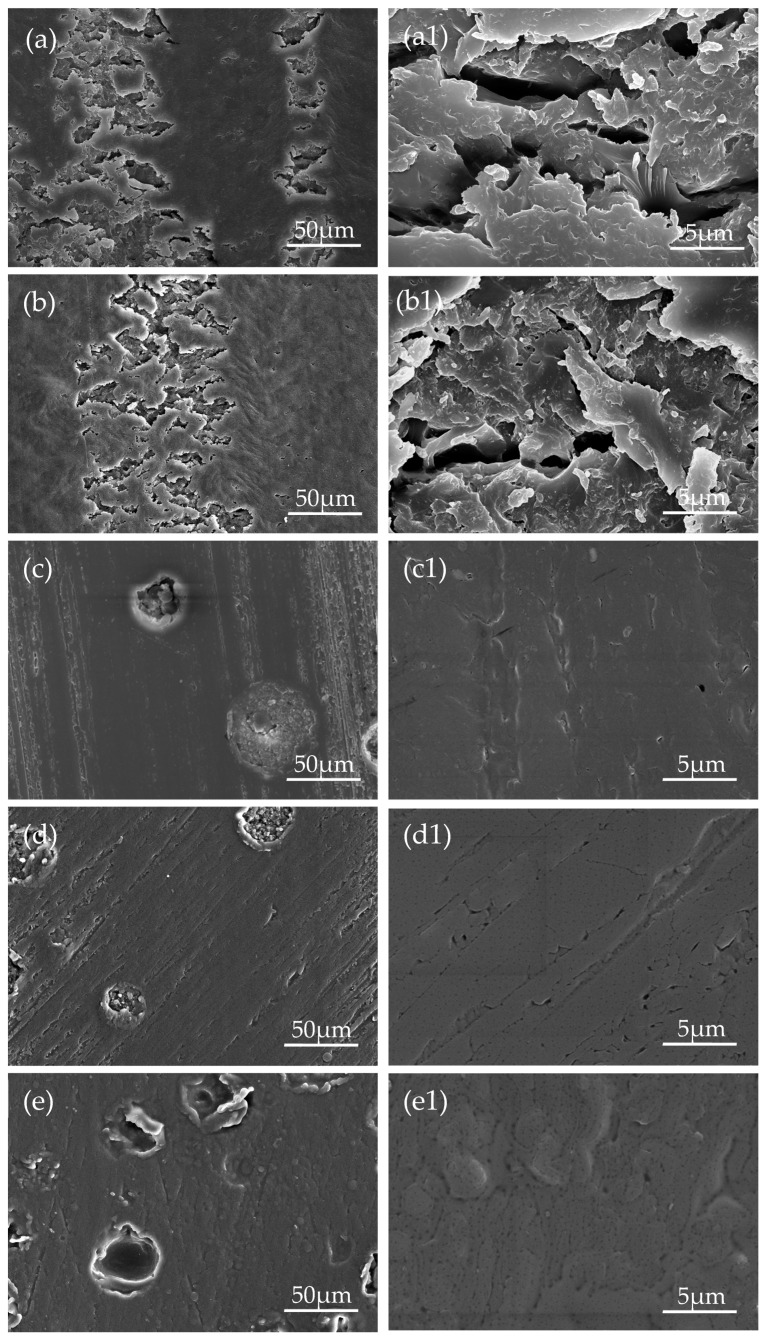
SEM micrographs showing worn surfaces of samples slid against a GCr15 steel ball of 4mm in diameter in a linear path of 5mm in length for ~1800s at a sliding speed of 5 cm/s, under a load of 3N: (**a**) pure epoxy coating, (**b**–**e**) coating with 5, 10, 15 and 20 wt.% microcapsules, and the respective partial enlargement (**a1**, **b1**, **c1**, **d1**, and **e1**).

The self-lubricity of the coating with different concentrations of microcapsules was evaluated by a friction wear test. Figure 10 shows the evolution of friction coefficients with sliding times for the pure epoxy coating and coatings with different concentrations of microcapsules, under dry sliding against GCr15 steel balls. The corresponding three-dimensional white light interferogram and scanning electron micrograph are shown in Figure 11 and Figure 12.

The friction coefficient of pure epoxy coating increases during the initial period and then gradually stabilizes, around 0.673. During the running-in period, the surface microprotrusions are worn away, which increases the contact area and causes more serious interlock behavior, resulting in an augment in the friction coefficient at initial. After addition of 5% microcapsules, the friction record is similar to pure epoxy coating, but exhibited apparent fluctuations. As the addition of microcapsules are 10, 15, and 20 wt.%, the friction coefficient of the coatings remained basically unchanged with sliding time, and is very low at ~0.07. The coating by incorporating 5, 10, 15, and 20 wt.% microcapsules affords 3.36%, 87.52%, 90.11%, and 90.65% friction coefficient reduction, respectively. The addition of LO microcapsules (in 10 wt.%) show stronger self-lubricating effects than HMDI (78.9%) [18] and tung oil (17.3%) [30]. Consequently, coatings exhibited excellent abrasion resistance as the content of microcapsules is increased to 10 wt.% or more.

**Figure 13:**
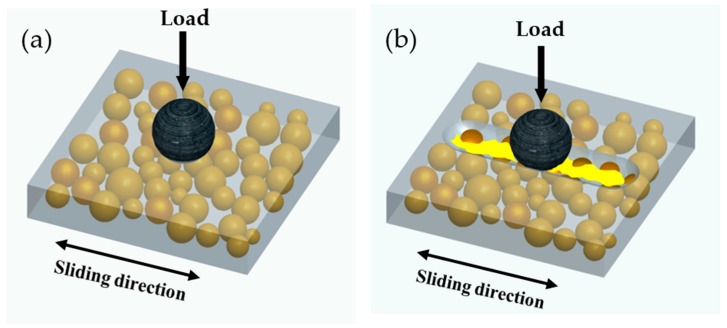
Schematic of the self-lubricating process: (**a**) Self-lubricating coating with microcapsules containing linseed oil slid against a steel ball under a load. (**b**) Damage of the coating; release of the encapsulated linseed oil at the friction interface.

The linear wear tracks gradually become shallower follow by the increase of microcapsules content as shown in Figure 11. Wear scars are basically invisible when the microcapsule content reached 10 wt.% or more, which was consistent with the tendency of friction coefficient. The morphological observation of the worn surface of all samples by SEM revealed that pure epoxy coating and the coating with 5 wt.% microcapsules exhibit fatigue wear characteristics. There are distinct grooves, which are filled with a large area of microcrack network, formed by the removal of materials along the sliding direction; the microcracks are generally perpendicular to the sliding direction. This is caused by repeated sliding of the GCr15 ball under load resulting in cyclic stress concentration on the surface of the coating. When the addition amount microcapsules less than 5 wt.%, the LO is not sufficient to increase the abrasion resistance of the coating. The coatings containing 10, 15, and 20 wt.% shows the relatively smooth and flat wear surface without visible microcracks and wear debris, indicating only slight adhesive wear. The aforementioned phenomenon was mainly attributed to the LO releasing of microcapsules after being worn out during the wear process. The LO forms a transfer film between the sample and the friction pair due to physical adsorption, which separates the friction interfaces that contact each other and reduces the shear resistance. Figure 13 shows a schematic of the self-lubricating process. Therefore, the coatings with microcapsules exhibit self-lubricating properties and become superior with increasing content.

## 4. Conclusions

In this study, the corrosion resistance and friction wear performances of epoxy composites with different contents of PU microcapsules containing bifunctional LO were systematically investigated. The coating is embedded with LO microcapsules exhibited excellent self-healing corrosion resistance in the EIS and salt spray tests; the corrosion resistance of the healing film formed within the scratched area was positively correlated with the microcapsules content. Furthermore, excellent self-lubricating properties of the epoxy resin with 10 wt.% microcapsules or more have also been confirmed. The epoxy coating shows 86.8% friction coefficient reduction after the concentration of microcapsules reached 10 wt.%. These results indicate that the addition of PU microcapsules containing LO is an available approach to improve the corrosion resistance and the friction wear performances of the epoxy composites. It heralds the enormous potential of microcapsules encapsulating LO in the field of bifunctional coating for self-healing and self-lubrication.

## Figures and Tables

**Table 1:** Circuit model parameters for the scratched coatings with 5, 10, 15, and 20 wt.% microcapsules at 0 and 3 days exposure time to 3.5% NaCl solution.

Time	Microcapsules Content (wt.%)	*R_healing_* (Ω•cm^2^)	*R_ct_* (Ω•cm^2^)	*Q_healing_*		*Q_dl_*		*W*
				*T* (F•cm^−2^s^P−1^)	*P*	*T* (F•cm^−2^s^P−1^)	*P*	*R* (Ω•cm^2^)
0 day	0	-	3.62 × 10^6^	-	-	2.39 × 10^−8^	0.756	-
	5	6.96 × 10^10^	-	2.01 × 10^−11^	0.964	-	-	-
	10	6.38 × 10^10^	-	2.90 × 10^−11^	0.896	-	-	-
	15	9.43 × 10^10^	-	2.36 × 10^−11^	0.950	-	-	-
	20	1.27 × 10^11^	-	3.20 × 10^−11^	0.922	-	-	-
3 days	0	-	6.59 × 10^4^	-	-	2.57 × 10^−5^	0.729	-
	5	17740	3.18 × 10^6^	1.83 × 10^−7^	0.358	2.99 × 10^−8^	0.031	8.17 × 10^5^
	10	9.42 × 10^6^	1.58 × 10^9^	3.74 × 10^−11^	0.937	3.44 × 10^−9^	0.683	-
	15	4.03 × 10^7^	1.35 × 10^11^	3.33 × 10^−11^	0.940	4.53 × 10^−9^	0.392	-
	20	6.69 × 10^10^	-	4.64 × 10^−11^	0.907	-	-	-
